# Can COVID-19 Vaccines Induce Premature Non-Communicable Diseases: Where Are We Heading to?

**DOI:** 10.3390/vaccines11020208

**Published:** 2023-01-17

**Authors:** Altijana Hromić-Jahjefendić, Debmalya Barh, Vladimir Uversky, Alaa A. Aljabali, Murtaza M. Tambuwala, Khalid J. Alzahrani, Fuad M. Alzahrani, Saleh Alshammeri, Kenneth Lundstrom

**Affiliations:** 1 Department of Genetics and Bioengineering, Faculty of Engineering and Natural Sciences, International University of Sarajevo, Hrasnicka Cesta 15, 71000 Sarajevo, Bosnia and Herzegovina; 2 Department of Genetics, Ecology and Evolution, Institute of Biological Sciences, Federal University of Minas Gerais, Belo Horizonte 31270-901, Brazil; 3 Institute of Integrative Omics and Applied Biotechnology (IIOAB), Nonakuri, Purba Medinipur 721172, India; 4 Department of Molecular Medicine and USF Health Byrd Alzheimer's Institute, Morsani College of Medicine, University of South Florida, Tampa, FL 33612, USA; 5 Department of Pharmaceutics and Pharmaceutical Technology, Faculty of Pharmacy, Yarmouk University, P.O. Box 566, Irbid 21163, Jordan; 6 Lincoln Medical School, Brayford Pool Campus, University of Lincoln, Lincoln LN6 7TS, UK; 7 Department of Clinical Laboratories Sciences, College of Applied Medical Sciences, Taif University, P.O. Box 11099, Taif 21944, Saudi Arabia; 8 Department of Optometry, College of Applied Medical Sciences, Qassim University, Buraydah 51452, Saudi Arabia; 9 PanTherapeutics, Route de Lavaux 49, CH1095 Lutry, Switzerland

**Keywords:** SARS-CoV-2, COVID-19 vaccines, premature NCDs, vaccine hesitancy

## Abstract

According to the WHO, as of January 2023, more than 850 million cases and over 6.6 million deaths from COVID-19 have been reported worldwide. Currently, the death rate has been reduced due to the decreased pathogenicity of new SARS-CoV-2 variants, but the major factor in the reduced death rates is the administration of more than 12.8 billion vaccine doses globally. While the COVID-19 vaccines are saving lives, serious side effects have been reported after vaccinations for several premature non-communicable diseases (NCDs). However, the reported adverse events are low in number. The scientific community must investigate the entire spectrum of COVID-19-vaccine-induced complications so that necessary safety measures can be taken, and current vaccines can be re-engineered to avoid or minimize their side effects. We describe in depth severe adverse events for premature metabolic, mental, and neurological disorders; cardiovascular, renal, and autoimmune diseases, and reproductive health issues detected after COVID-19 vaccinations and whether these are causal or incidental. In any case, it has become clear that the benefits of vaccinations outweigh the risks by a large margin. However, pre-existing conditions in vaccinated individuals need to be taken into account in the prevention and treatment of adverse events.

## 1. Introduction

The development of effective vaccines has been necessary for slowing down and stopping the COVID-19 pandemic. Several COVID-19 vaccines have received emergency use authorization (EUA) and some have already been approved. Vaccines based on cutting-edge technologies for the delivery of genetically engineered mRNA molecules (Pfizer/BioNTech, Moderna) have been applied for the in vivo production of the SARS-CoV-2 spike (S) protein [1,2,3]. Vaccines based on nonreplicating recombinant adenovirus vectors (AstraZeneca/University of Oxford, Johnson & Johnson, Gamaleya Research Institute) have also been used for the expression of the S protein [3,4,5]. Whole SARS-CoV-2 particles have also been the subject of vaccine development, with complete inactivated vaccines being made available (Sinovac Biotech) [4]. Although the above-mentioned vaccines have prevented severe COVID-19 symptoms, they have potentially been associated with post-vaccination adverse events [5], although at low numbers. 

The WHO defines non-communicable diseases (NCDs) as long-lasting chronic diseases caused by a combination of genetic, physiological, environmental, and behavioral factors [6]. In rare cases, vaccines have been hypothesized to induce adverse events that may contribute to the development of NCDs. Cardiovascular diseases (CVDs), cancers, chronic respiratory diseases, diabetes, and neurological diseases (NDs) are the most common forms of NCDs. More than three-quarters of global NCD fatalities (31.4 million) occur in low- and middle-income countries. The Centers for Disease Control and Prevention (CDC) reports that NCDs are the most common cause of mortality throughout the world. As a sobering reminder of the relevance of NCDs in outbreak planning and response, NCDs and associated risk factors increase the chance of hospitalization or mortality from COVID-19 in all age groups [7]. 

The majority of NCDs associated with COVID-19 vaccines account for: (1) CVDs including hypertension, myocarditis, infarctions, thromboembolic events, and arrhythmia [3,8,9,10]; (2) premature obesity and diabetes [11,12,13,14,15]; (3) acute kidney disorders (AKDs) [16]; and (4) premature neurodegeneration and mental disorders [17,18,19,20,21], and autoimmune spectrum disorders including vaccine-induced immune thrombotic thrombocytopenia (VITT) and leukocytoclastic vasculitis [22,23]. Other systemic adverse reactions such as fever, headache, widespread fatigue, chills, arthralgia, muscular pain, and nausea [24] are transient and not discussed in this review. Moreover, concerns have been raised related to the effect of vaccinations on infertility, reproductive health, and lactation [25,26,27].

Previously, the impact of SARS-CoV-2 on persons with congenital and genetic disorders [28] and COVID-19-associated comorbidities have been described [29]. Here, we investigate the possible association between COVID-19 vaccines and the onset of NCDs and evaluate any effect on reproductive health and fertility caused by COVID-19 vaccines. Importantly, as adverse events after vaccination are rare, very few cases have been detected even in clinical trials with several thousand participants. For this reason, case reports have represented the main source for adverse events occurring after vaccination. However, data from case reports do not carry the same weight as data from randomized clinical trials.

## 2. Literature Search

**Figure 1:**
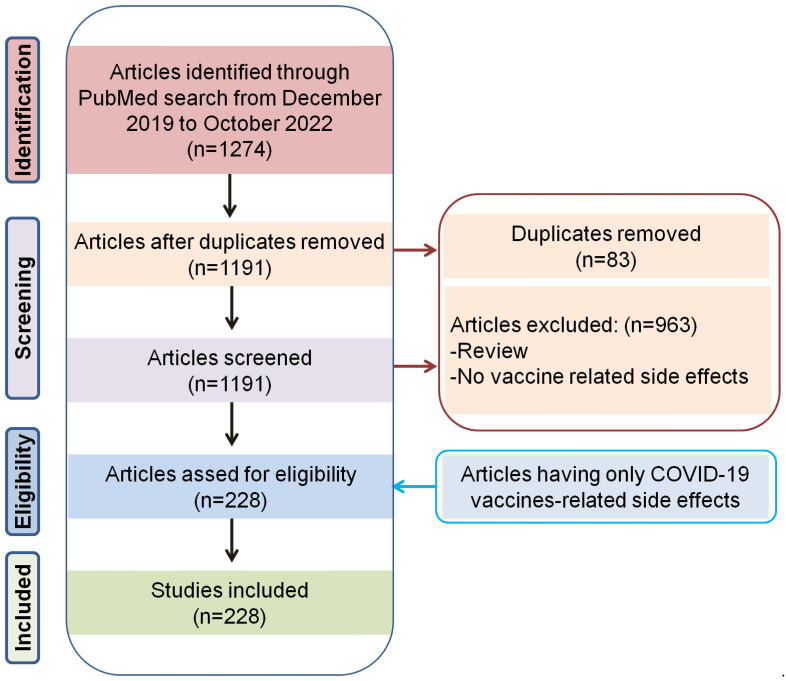
PRISMA flow diagram of literature search and article selection to develop the key sections of this review.

The advanced search option of the PubMed [30] literature database was carried out from December 2019 to October 2022 using (keywords in the title) the following keywords: (COVID-19 [Title]) AND (Vaccine [Title]) AND (cardiac [Title]), (COVID-19 [Title]) AND (Vaccine [Title]) AND (kidney [Title]), (COVID-19 [Title]) AND (Vaccine [Title]) AND (disease [Title]), (COVID-19 [Title]) AND (Vaccine [Title]) AND (mental [Title]), and so on, to retrieve the relevant articles. We used more than 70 different conditions as key words under cardiovascular, metabolic, renal, mental, neurological, and reproductive diseases. Once the articles were retrieved, an abstract-level manual curation was performed following the PRISMA method to select the final articles for the review. A total of 1274 articles were retrieved and based on the PRISMA method, finally, 228 articles describing COVID-19-vaccine-related side effects were included to develop the main sections of the review (Figure 1). We also used additional literature to describe various other basic information in this review.

## 3. COVID-19 Vaccines and Premature Diabetes

Diabetes and COVID-19 have a bidirectional relationship. In addition to causing new onset or worsening pre-existing diabetes, diabetes and hyperglycemia are linked to a worse prognosis in COVID-19 patients. A small fraction of individuals diagnosed with COVID-19, many of whom did not have a previous history of diabetes mellitus (DM), have been found to experience extreme hyperglycemia and acute hyperglycemic emergencies such as diabetic ketoacidosis (DKA) based on case reports [11,12,13,14,31], and/or hyperosmolar hyperglycemic syndrome (HHS), with 91 cases of DKA and 19 cases of DKA/HHS reported in clinical trials [11,12,13,14,31]. The co-existence of COVD-19 and diabetes is a two-way street. Individuals with diabetes and hyperglycemia have shown a poorer prognosis of COVID-19, but SARS-CoV-2 infections can also play a role in the development of new diabetes or the worsening of pre-existing diabetes. Extreme hyperglycemia and acute hyperglycemic crises such as DKA have been observed in persons diagnosed with COVID-19, many of whom did not have a previous history of diabetes. Pre-existing DM has been linked to severe COVID-19 such as acute respiratory distress syndrome (ARDS), septic shock, and an approximately threefold increased risk of in-hospital death based on a meta-analysis of 83 eligible clinical trials [32] and a retrospective cohort study in 1226 hospitalized COVID-19 patients [31], respectively. 

Significant changes in inflammatory markers such as C-reactive protein (CRP), procalcitonin, D-dimer, and lymphopenia have been described in people with pre-existing type 2 diabetes (T2DM), and notably in poorly managed T2DM with hyperglycemia [33]. Not all of the mechanisms linking SARS-CoV-2 infection and hyperglycemia have been elucidated; these may include direct infection of the pancreatic islets by the virus or other routes involving the inflammatory or stress responses to the virus [12,34,35,36]. As the S protein utilizes the cellular ACE2 receptor in essential organs, SARS-CoV-2 can infect and replicate in endocrine and exocrine pancreatic cells as part of its pathogenesis [37]. Moreover, the presence of ACE2 on the surface of lung alveolar epithelial cells and enterocytes of the small intestine suggested a possible route of entry for SARS-CoV and provided some insights into its pathogenesis [38]. 

In a study in mice, the absence of ACE2 led to impaired glucose homeostasis, causing increased susceptibility to T2DM [39]. In the case of SARS-CoV, ACE2-receptor-based destruction of pancreatic beta cells results in acute diabetes [35]. As pancreatic beta cells possess ACE2 receptors, they are vulnerable to SARS-CoV-2 infections [37]. The SARS-CoV-2 susceptibility of pancreatic cells suggests that they can be destroyed upon virus entry [40]. 

COVID-19 vaccines may have worsened diabetes control in individuals with known cases of T1DM and T2DM and caused a hyperglycemic crisis although it is unclear whether it is a question of a temporal or causal effect [13,14,41,42]. One case each of DKA has been reported after vaccinations with the adenovirus ChAdOx1 nCoV-19, the inactivated whole virus Covaxin [43], and the mRNA-1273 [11] vaccines in case reports. Moreover, in a case series, three cases of hyperglycemia were reported after vaccination with the ChAdOx1 nCoV-19 vaccine [37] and one case of HHS after BNT162b2 vaccination [44,45]. Although the pathophysiology of the link between SARS-CoV-2 infection and hyperglycemia is not fully known. It may be caused by a direct SARS-CoV-2 infection of the pancreatic islets or by additional pathways involving inflammatory or stress reactions to the virus [12,34,36]. The production of anti-insulin hormones (cortisol, catecholamines, and growth hormone) as a result of an inflammatory and/or immunological reaction to the vaccine may cause acute stress and lead to a hyperglycemic crisis [44]. Additionally, concerns have been raised over the possibility that infection or newly developed vaccines could cause autoimmunity via cross-reactivity [46]. Cross-reactivity happens when a pathogenic protein and a self-tissue protein show high amino acid sequence similarity [47]. According to this mechanism, antibodies elicited against SARS-CoV-2 binding to proteins in human tissue cause autoimmune reactions [46]. As a result, COVID-19-vaccine-induced SARS-CoV-2-specific antibodies may interact with human pancreatic tissues resulting in autoimmune-disease-like T1DM in susceptible individuals [48]. For example, GAD-65 [46], one of the key antigens involved in immune-mediated T1DM [49], shows a strong reaction against SARS-CoV-2 S antibodies.

In a multi-center prospective clinical study, the short-term impact of COVID-19 immunization was explored by measuring glucose levels by continuous glucose monitoring (CGM) in individuals with T1DM and T2DM, supporting the assumption that hyperglycemia occurrence is relatively uncommon [46]. A total of 161 individuals participated in the multi-center prospective Immune Response to COVID-19 Vaccination in People with Diabetes Mellitus—COVAC-DM Study from April to June 2021. The results showed that COVID-19 vaccination itself did not affect the ability of diabetics to control their blood sugar levels [50]. In a clinical trial with 135 participants, 70 children and adolescents with T1DM were vaccinated with the BNT162b2 vaccines, with 65 individuals belonging to the control group, showing good safety profiles, and the vaccination did not cause acute glucose imbalance [51,52]. In another clinical trial, 39 adolescents with T1DM were vaccinated with either the BNT162b2 or the mRNA-1273 vaccine, which was safe and was not associated with a perturbation in glycemic control [48]. Moreover, there was no evidence of acute diabetic complications after either the first or second doses of the BNT162b2 mRNA or CoronaVac inactivated vaccines in a self-controlled case series analysis of T2DM patients in Hong Kong [52,53]. Additionally, subgroup analysis of individuals with hemoglobin A1c (HbA1c) levels <7% or ≥7% did not show significant differences in the risk of adverse events following immunization. In a phase III trial, no unexpected patterns related to safety and tolerability were detected in 454 T1DM patients [50]. Although transient mild adverse events were common, only one serious adverse event was detected, which was considered unrelated to the vaccination.

However, in a retrospective study of 96 T1DM patients, blood glucose levels showed perturbation after the first dose of COVID-19 vaccination, which was more prominent when the HbA1c was lower [54,55]. Moreover, a study in 97 TD1M patients vaccinated with either the BNT162b2 or the ChAdOx1 nCoV-19 vaccine showed perturbation of interstitial glucose, which was enhanced by oral hypoglycemic drugs [56]. In a case study, a 55-year-old male heart transplant recipient with mild diabetes who received the Ad26.COV2.S vaccine showed a rapid and severe worsening of his diabetes [57]. Multi-dosing insulin treatment improved his clinical condition and controlled glycemia. However, the case demonstrated the importance of closely monitoring diabetes patients after vaccination. 

Following mRNA vaccinations, cases of newly developed T1DM have been documented [48,58,59]. According to a case report, a 51-year-old Japanese woman was diagnosed with acute-onset of T1DM and DKA six weeks after receiving the first dose of the mRNA-1273 vaccine [58]. Antigen genotyping from leukocytes identified DRB1*09:01-DQB1*03:03 homozygosity, common among T1DM patients in Japan. However, prospective large-scale clinical trials need to confirm the causal relationship between vaccinations and the onset of T1DM. In another case, a 36-year-old female developed hyperglycemia, excessive thirst, frequent urination, heart palpitations, lack of appetite, and exhaustion three days after receiving the first dose of the BNT162b2 vaccine [59]. Ten days after the first vaccine administration, she showed DKA and was diagnosed with D1TM. She carried the DRB1*0405-DQB1*0401 haplotype associated with D1TM in Japan. The case suggested that even without a prior history of T1DM, individuals genetically pre-disposed to D1TM should be carefully surveyed after COVID-19 vaccinations. Furthermore, a 73-year-old Japanese woman was given two doses of the mRNA-1273 vaccine [60]. Her glycemic control began to worsen four weeks after the second vaccination, and eight weeks later, she was diagnosed with onset of T1DM showing a strong autoantibody response. She carried the disease-susceptible haplotype DRB1*04:05:01-DQB1*04:01:01. In another case study, four individuals were diagnosed with T1DM a few weeks after immunization with the BNT162b2 vaccine [48]. However, a rapid decrease in insulin requirement was seen in all patients, and the need for insulin therapy disappeared in three patients during the follow-up period. 

In March 2022, a healthy, completely asymptomatic 50-year-old man was diagnosed with fulminant T1DM (FT1DM) with total and irreversible islet damage six days after receiving the COVID-19 inactivated vaccine (CoronaVac^®^) [61]. Laboratory tests showed hyperglycemia, ketosis, metabolic acidosis, and near-normal HbA1c levels. The patient had no family history of T1DM, although his 73-year-old mother had T2DM. After receiving the vaccine, however, the patient suddenly developed mild fever, excessive thirst, and frequent urination. Although the precise mechanism for the FT1DM pathogenesis was unclear, genetic susceptibility and autoimmunity might have contributed to the onset of FT1DM [61]. Finally, a 45-year-old Japanese woman developed FT1DM eight days after receiving the BNT162b2 vaccine [62]. She was in good health and exhibited no signs of viral infection before the immunization. However, she carried the DRB1*04:05 and DQB1*04:01 alleles, associated with T1DM in Japan. Although the onset of FT1DM occurred relatively soon after the COVID-19 vaccination, the causal relationship could not be established [62].

## 4. COVID-19 Vaccines and Premature Cardiovascular Diseases (CVDs)

### 4.1. COVID-19 Vaccines and Premature Hypertension

Several comorbidities and biomarkers related to COVID-19 are independent predictors of serious illness and unfavorable outcomes [63,64,65]. Since the beginning of the pandemic, the relationship between hypertension and COVID-19 has been debated. In a review, 14 publications were analyzed to investigate a possible association between hypertension and COVID-19 and the impact of hypertension on patient outcomes [66]. According to this review, one of the most prevalent comorbidities among COVID-19 patients is arterial hypertension. The involvement of ACE2 in SARS-CoV-2 infection and its role in several cardiovascular and immune pathways led to the hypothesis that hypertension may play a role in the development of COVID-19 [66]. In COVID-19 patients, hypertension was linked to a 2.5-fold higher risk of both increased disease severity and mortality [67]. 

The retrospective screening was used to examine the impact of COVID-19 on blood pressure in 211 COVID-19 patients admitted to the Parkhayat Kutahya hospital in Turkey during the short-term follow-up [68]. Compared to pre-COVID-19 levels, both systolic and diastolic blood pressures were significantly elevated after COVID-19. After an average of 31.6 ± 5.0 days, 18 individuals showed signs of new-onset hypertension (*p* < 0.001). These results suggest that exposure to SARS-CoV-2 increases both systolic and diastolic blood pressure and may lead to the development of hypertension in previously healthy individuals [68]. 

There are only a few reported incidences of hypertension after vaccination with COVID-19 mRNA vaccines [66]. In a case study, a 71-year-old woman collapsed three days after receiving the first dose of the mRNA-1273 vaccine [67]. She had high blood pressure and an intracranial hemorrhage in the left basal ganglia, but no thrombocytopenia and no abnormalities in blood tests. Despite treatment with clonidine hydrochloride and furosemide, she passed away nine days after the initial event [69]. In a case series study, stage III hypertension was reported in nine patients only minutes after receiving either the BNT162b2 or the mRNA-1273 vaccine [68]. The nine patients had a median age of 73, indicating that older people with a history of hypertension or cardiovascular comorbidities must have their blood pressure under control prior to immunization and be well monitored afterward [70].

A total of 8276 hypertensive incidents, 108 hypertensive crises, and 91 cases of hypertensive urgency were reported to the Vaccine Adverse Event System (VAERS) in the US following COVID-19 vaccinations as of July 2022 [71]. Moreover, 941 cases of hypertension, 14 cases of hypertensive crisis, and 4 cases of hypertensive urgency were found in a case series of people who had received the ChAdOx1 nCoV-19 vaccination [72]. After vaccination, the prevalence of hypertension may vary depending on age and gender. There were also six individuals out of 113 who demonstrated an average elevation in systolic or diastolic blood pressure of more than 10 mm Hg between days five and ten after receiving two doses of the BNT162b2 vaccine [73].

In another study, 797 healthcare professionals were enrolled for blood pressure measurement at home following immunization with the BNT162b2 vaccine [74]. Stage II and III hypertension were detected in three and four participants, respectively. The increase in blood pressure occurred at the end of the first week after the first dose. It lasted for 3–4 days and was repeated after the second vaccination. Only one person, who had a history of cardiovascular disease, needed hospitalization. In conclusion, the COVID-19 vaccination seemed to cause only rare, benign, and transient increases in blood pressure, which led to recommending blood pressure monitoring only to individuals with a high risk of CVD [74]. 

In the case of vaccination with the BNT162b2 vaccine, 4201 cardiovascular adverse events were recorded, 400 cases after receiving the mRNA-1273 vaccine, and 262 cases after receiving the ChAdOx1 nCoV-19 vaccine, all according to the worldwide pharmacovigilance database VigiBase [72]. The adverse events were associated with acute myocardial infarction, cardiac arrest, and circulatory collapse in individuals older than 75 years. Adverse events such as hypertension, severe hypertension, supraventricular tachycardia, sinus tachycardia, and palpitations were neither associated with age nor gender. As the world population is already predisposed to conditions such as hypertension, coronary artery disease, arrhythmia, etc., COVID-19 vaccinations cannot be solely attributed to these adverse events and the causality needs to be investigated in large clinical trials [72]. 

### 4.2. COVID-19 Vaccines and Thromboembolic Events

Mass COVID-19 vaccinations have revealed some severe adverse events in a small number of subjects, the origins of which are not well understood, yet [75]. The free-floating spike proteins released from degraded vaccine-targeted cells circulating in the blood may attach to ACE2 receptors on other cells, which can lead to internalization and degradation of ACE2. Eventually, the loss of ACE2 activity could contribute to adverse events including platelet aggregation, thrombosis, and inflammation [76]. 

Thrombotic thrombocytopenia has been detected in individuals vaccinated with adenovirus-based COVID-19 vaccines [8,77] and also with the BNT162b2 and mRNA-1273 mRNA vaccines [78]. Although the standardized morbidity ratio for thromboembolic events was 1.97-fold greater for recipients of the ChAdOx1 nCov-19 vaccine compared to the general population, the absolute risks of venous thromboembolic events are small [78]. The immunological thrombotic thrombocytopenia potentially associated with the ChAdOx1 nCoV-19 vaccine is clinically similar to severe heparin-induced thrombocytopenia when platelet-activating antibodies against platelet factor 4 (PF4) are present [79,80]. For instance, the development of platelet-activating antibodies against PF4 in ten patients who experienced one or more thrombotic events was associated with the ChAdOx1 nCoV-19 vaccine [81]. In another study, 22 patients were evaluated for thrombosis and thrombocytopenia, and one patient for thrombocytopenia and very high levels of D-dimer temporally associated with one dose of the ChAdOx1 nCoV-19 vaccine [79]. Most of the clots were found in the veins of the brain, but some were found in the arteries, and a few were found in the lungs as pulmonary emboli. Since these individuals did not suffer from any pre-existing prothrombotic medical conditions, immunization may be responsible for the onset of the harmful PF4-dependent syndrome [82]. The Ad26.COV2.S vaccine has been used in more than 7 million people throughout the world but only six occurrences of cerebral venous sinus thrombosis with thrombocytopenia have been reported [83]. In addition, deep vein thrombosis was reported in one person in a randomized double-blind, placebo-controlled phase III trial with the Sputnik V vaccine in 14,964 volunteers [84]. Related to COVID-19 mRNA vaccines, cases of central nervous system thrombosis have been reported after the administration of the BNT162b2 and mRNA-1273 vaccines [85]. There are several hypotheses regarding the origins of VITT, including interactions between the SARS-CoV-2 S protein and various C-type lectin receptors, heparan sulfate proteoglycans (HSPGs), and the CD147 receptor, between soluble splice variants of the S protein, and between adenovirus vectors and the CD46 receptor or PF4 antibodies [86].

The exact mechanisms that might cause a hypercoagulable state following vaccination remain unknown. The ChAdOx1 nCoV-19 vaccine has been linked to five cases of severe venous thromboembolism, four of which were cerebral venous thrombosis, according to a case study [87]. Thrombotic thrombocytopenic purpura and immune thrombocytopenia were ruled out since all patients improved after transfusions and there was no evidence of hemolysis. Immunoglobulin G antibodies against PF4-polyanion complexes were elevated in all patients, indicating VITT rather than heparin-induced thrombocytopenia [87].

### 4.3. COVID-19 Vaccines and Premature Cardiac Arrhythmia

Although one case of paroxysmal ventricular arrhythmia was recorded in a phase III clinical trial for the BNT162b2 vaccine in 21,720 volunteers, no causal association could be established [3]. Importantly, the development of ventricular arrhythmias has been linked to cardiac inflammation [88]. In a case study, three people who tested positive for COVID-19 after receiving the BNT162b2 immunization had tachycardia [89]. Additionally, postural orthostatic tachycardia, a type of arrhythmia, previously detected in persons vaccinated with the human papillomavirus vaccine Gardasil [90], was detected in a healthy person 6 days after receiving the first dose of the BNT162b2 vaccine in a case study [91]. According to one theory, the immunological response to adrenergic receptors could impair vasoconstriction, which therefore results in postural tachycardia [92]. Poor vasoconstrictive response and subsequent orthostatic tachycardia in the upright position have been linked to impaired baroreflex function and diminished vasopressor response to plasma angiotensin-II [89]. On the other hand, the risk of arrhythmia increases by a factor of 3.83 when COVID-19 is present [93].

### 4.4. COVID-19 Vaccines and Premature Myocardial Infarction

Myocardial infarction has been reported in persons receiving mRNA-based, adenovirus-based [94], and inactivated whole SARS-CoV-2 [43] vaccines. In a multi-national cohort study on the prevalence of vaccine-induced adverse effects, the myocardial infarction risk increased with age after COVID-19 vaccination [95]. However, no conclusive evidence of an association between vaccination and myocardial infarction in any of the studies could be established.

### 4.5. COVID-19 Vaccines and Premature Takotsubo Cardiomyopathy

Takotsubo cardiomyopathy has been linked to one case of immunization with the mRNA-1273 [96] vaccine and another one with the theChAdOx1 nCoV-19 [97] vaccine in case reports. Notably, neither patient showed any triggering of viral, inflammatory, mental, or physical factors, and both recovered after therapy [98]. Additionally, based on a systematic literature search, 10 cases of post-vaccination Takotsubo cardiomyopathy were detected in 10 studies confirming that the incidences are rare but life-threatening [96].

### 4.6. COVID-19 Vaccines and Premature Myocarditis

Vaccines against, for example, smallpox have previously shown an association with acute myocarditis, especially in young males, although cases have remained rare [99]. Similarly, vaccination, particularly with mRNA-based COVID-19 vaccines, has revealed the presence of cases of myocarditis after global mass vaccinations [100]. Rare complications have occurred in young adult and adolescent males in the form of myocarditis/pericarditis at rates of 12.6 cases per one million doses of second-dose mRNA COVID-19 vaccines [101]. Patients with myocarditis typically had chest discomfort, high levels of cardiac troponin, and abnormal electrocardiograms. Vaccine-induced myocarditis is a complex process that includes, but is not limited to, the activation of immunologic pathways, immunological responses to mRNA, activation of dysregulated cytokine production [101]. MRI scans indicated that patient symptoms were resolved in nearly all situations. For instance, 23 incidences of myocarditis were found in US military members who had been vaccinated with an mRNA-based COVID-19 vaccine [102]. After the second mRNA vaccine dose, the patients showed the classic symptoms of myocarditis, including severe chest pain, increased troponin levels, and other cardiovascular symptoms. All patients made a full recovery. Furthermore, it was noted that myocarditis instances were extremely rare because over 2 million doses of mRNA vaccines have already been administered to US military troops. The pharmacovigilance database VigiBase was analyzed, which had a total of 25,728,751 reports on adverse medication reactions, including 8664 reports on myocarditis, of which 1251 were possibly related to mRNA-based COVID-19 vaccines [103]. Myocarditis has been linked to the mRNA-1273 and BNT162b2 vaccines but not to other COVID-19 vaccines. Myocarditis occurred in 2.13 people out of every 100,000 vaccinated in a large study of a healthcare organization in Israel [104]. Males aged 16 to 29 showed the highest prevalence, with 10.69 new cases per 100,000 men after receiving the BNT162b2 vaccine. The severity of the symptoms was rated as mild in 76% of the cases, moderate in 22%, and severe in one instance (cardiogenic shock). One patient had to be re-admitted to hospital 83 days after the commencement of myocarditis, while another patient died of undetermined causes after being released. Ten out of 14 individuals were found to have detectable left ventricular dysfunction at the time of their hospital discharge. Following additional testing, heart function returned to normal in five of the ten patients.

Cardiovascular symptoms, including myocarditis and pericarditis occurrences, were assessed in a prospective cohort study of 301 adolescents and young adults who had received the second dose of the BNT162b2 vaccine [105]. Heart problems were the leading cause of hospitalization, with tachycardia (7.64%), dyspnea (6.64%), heart palpitations (4.32%), chest pain (4.32%), and hypertension (7.32%). Myocarditis affected one patient, subclinical myocarditis was suspected in four others, and pericarditis in two. Myopericarditis seldom caused serious symptoms and always resolved itself within two weeks.

In the US, adolescents and young adults who were diagnosed with myocarditis 90 days after receiving COVID-19 mRNA vaccinations were recently subjected to follow-up surveillance research [106]. Among the 519 persons surveyed, 126 replied to patient questionnaires, 162 to healthcare provider surveys, and 231 to both. Of the 393 individuals evaluated for health treatment, 320 were considered recovered from myocarditis. In addition, 104 patients out of 393 were provided daily treatment for myocarditis. Among the 249 persons who completed the survey's quality-of-life section, four (2%) reported difficulties with self-care, 13 (5%) with mobility, 49 (20%) with doing daily tasks, 74 (30%) with pain, and 114 (46%) with depression. Most patients with myocarditis had normal troponin concentrations, echocardiograms, electrocardiograms, exercise stress test findings, and ambulatory rhythm. Overall, the majority of persons were considered recovered and had equivalent quality-of-life indicators to those before the pandemic.

As more or less all studies have demonstrated, the pattern of COVID-19-vaccine-related myocarditis is similar to those of other viral infections, showing a higher frequency among adolescent and young adult males [103,107]. This is potentially related to the elevated testosterone effect on interleukin-1 (IL-1) production [108]. However, most importantly, the risk of myocarditis and hospitalization in vaccinated individuals in this age group is lower than for unvaccinated COVID-19 patients [109]. In summary, as approximately half a billion individuals had received at least one dose of a COVID-19 vaccine at the time of analysis, it is obvious that there is a clear benefit over risk for a vaccination strategy [103]. 

## 5. COVID-19 Vaccines and Premature Acute Kidney Disease (AKD)

A few cases of acute kidney injury (AKI), acute kidney disease (AKD), proteinuria, edema, gross hematuria, and other renal adverse events have emerged since the introduction of COVID-19 mass vaccinations [109]. However, AKD cases after vaccinations are very rare. Moreover, affected patients have benefited from prompt treatment with steroids when symptoms such as hematuria, foamy urine, and edema are identified at an early stage.

In most reported cases, serum creatinine (Scr) levels and proteinuria improved after three months of therapy. A few cases of glomerulonephritis have been linked to inactivated COVID-19 vaccines, although the great majority of cases occurred after vaccination with mRNA- and adenovirus-based vaccines. A total of 53 cases of AKI, including six (11%) recurrence cases and 47 (89%) new kidney involvements, have been documented after COVID-19 vaccinations in case report studies [16,108,109,110,111,112,113,114,115,116,117,118,119,120,121,122,123,124,125,126,127]. The most frequent pathology was 13 (25%) cases of minimal change disease (MCD), 11 (21%) cases of IgA nephropathy (IgAN), eight (15%) cases of acquired thrombotic thrombocytopenic purpura (aTTP), and eight (15%) cases of anti-neutrophil cytoplasmic autoantibodies (ANCA). Additional diseases such as four cases of acute interstitial nephritis (AIN), four cases of membranous nephropathy (MN), three cases of anti-glomerular basement membrane (anti-GBM) nephritis, one case of granulomatous vasculitis, and one case of leukocytoclastic vasculitis were also diagnosed [16]. Enhanced Src levels were confirmed in only one patient and his kidney functions returned to normal within a week [125].

### 5.1. Induction of AKI after COVID-19 Vaccination

#### 5.1.1. COVID-19 Vaccines and Podocyte Damage

The relationship between intramuscular vaccination and the onset of MCD suggests that podocyte damage may be brought on by a cell-mediated immune response [114,128]. Following immunization, dendritic cells usually pick up vaccine antigens and then present them to T cell receptors on naïve T cells. As a result. effector T cells, specific for antigens, are activated reaching their peak 7 to 14 days after immunization. Cellular immune responses to viral particles can be detected within a week after infection, while T-cell activation is initiated up to two or three days earlier [114,129,130,131]. It appears that circulating substances generated by activated T lymphocytes are the primary cause of podocyte destruction. T-cell subsets are out of balance during the active stages of MCD and circulating CD8^+^ T-cells reduce the number of T cells, which is further worsened by cytokine-induced damage [132,133]. It is anticipated that mRNA vaccines induce stronger chemokine and cytokine responses than traditional vaccines, as well as greater CD8^+^ T- and CD4^+^ T-cell reactions and stronger antibody responses [134]. On the other hand, SARS-CoV-2 may enter kidney glomerular podocytes by binding to ACE2, which results in podocyte dysfunction [135]. The BSG/CD147 and ACE2 receptors have been identified as key mediators for the SARS-CoV-2 S protein binding leading to severe AKI.

#### 5.1.2. COVID-19 Vaccines and Increased Production of Anti-Neutrophil Cytoplasmic Autoantibodies (ANCAs)

In the case of COVID-19, an autoimmune reaction and ANCA-associated vasculitis (AAV) can be directly caused by the host's reaction to viral RNA [136,137,138]. In contrast to the first dose of COVID-19 mRNA vaccines, a stronger response from the innate immune system was detected after the second vaccine dose [139]. MPO-ANCA and PR3 autoantibodies were induced by the enhanced innate immune response after the second dose of the BNT162b2 mRNA vaccine. Leukocyte membranes contain toll-like receptors (TLRs) and are crucial for triggering inflammatory reactions, identifying viral antigens, and boosting immune system activation [140]. Autoimmunity may be induced in AAV by significant TLR2 and TLR9 activation [141]. TLR2 may also have been triggered by a strong and specific immunological response of immunodominant cytotoxic T-lymphocytes (CTLs) to the SARS-CoV2 protein [141]. As opposed to previous COVID-19 vaccinations, it has been shown that AAV is associated with COVID-19 mRNA vaccines [16]. However, further investigations are needed to confirm the mechanism of the association between autoimmunity and COVID-19 vaccines.

## 6. COVID-19 Vaccines and Neurological Adverse Events

In the context of neurological diseases, an increasing number of neurological symptoms and manifestations have been suggested to be associated with COVID-19 vaccines [142]. Due to mass vaccinations, some neurological conditions are expected [143]. For example, two patients experienced transverse myelitis after receiving the ChAdOx1 nCoV-19 vaccine in at the end of 2020, which raised concerns about neurological side effects related to COVID-19 vaccinations [143]. However, one of the cases was considered unrelated to vaccination as the person in question had pre-existing multiple sclerosis (MS), while the other case could be related to vaccination. In another study, neurological side effects of the BNT162b2 and ChAdOx1nCoV-19 vaccines were investigated and compared to individuals infected by SARS-CoV-2 in a population-based study of over 32 million people [144]. Although persons receiving the ChAdOx1nCoV-19 vaccine showed a higher risk of myasthenic diseases, Bell's palsy, and Guillain–Barré syndrome (GBS) and individuals vaccinated with the BNT162b2 vaccine had an increased risk of hemorrhagic stroke (1–7 days and 15–21 days after vaccination), there was a substantially higher risk of acute CNS demyelinating episodes such as encephalitis, myelitis, GBS, meningitis, myasthenic disorders, hemorrhagic stroke, Bell’s palsy, and subarachnoid hemorrhage in COVID-19 patients [144]. For example, while 38 excess cases of GBS per 10 million persons were detected in individuals receiving the ChAdOx1 nCoV-19 vaccine, a significantly higher number of 145 cases per 10 million people were seen in COVID-19 patients. In conclusion, even though a higher risk of neurological complications was associated with COVID-19 vaccinations, the risks are much higher in individuals contracting COVID-19 [144]. Hundreds of notifications for seizures, Bell's palsy, transverse myelitis, and GBS have been documented from the over 65 million doses of COVID-19 vaccinations administered in the UK through the Yellow Card COVID-19 vaccine adverse event reporting system [145]. Although reports continue to emerge, the statistics have not surpassed the predicted case rates, so far. 

### 6.1. COVID-19 Vaccine and Cerebral Venous Sinus Thrombosis

Cerebral venous sinus thrombosis (CVST) is the most significant vaccine-related neurological adverse event, and it typically occurs following vaccination with adenovirus-based COVID-19 vaccines [146]. Acute demyelinating polyneuropathy, acute disseminated encephalomyelitis, and acute transverse myelitis are all neurological complications that have arisen as a direct result of the molecular mimicry phenomenon. Herpes zoster reactivation has been documented after immunization after the first dose of the BNT162b2 vaccine [147]. Fortunately, as compared to the vast number of people throughout the world who have received COVID-19 vaccines, the incidences of serious neurological occurrences are extremely low [148,149]. In March 2021, there have been some rare cases of CVST in Europe following vaccination with the ChAdOx1 nCoV-19 vaccine [80]. For example, two young males developed severe thrombocytopenia and fatal CVST [78]. In another study, 23 patients experienced thrombosis and thrombocytopenia 6–24 h after vaccination with the ChAdOx1 nCoV-19 vaccine [82]. In a case study, five individuals vaccinated with the ChAdOx1 nCoV-19 vaccine experienced acute vascular events that were clinically recognized as abdominal vein thrombosis, arterial cerebral blood clots, CVST, and/or thrombotic microangiopathy [149]. All individuals exhibited significantly elevated D-dimer levels, decreased platelet counts, and the presence of PF4 autoantibodies. Based on adverse events notified under the Medical Dictionary for Regulatory Activities Term, 213 vaccinated persons developed CVST, and 107 (57%) of the 187 ChAdOx1 nCoV-19-vaccinated individuals also developed thrombocytopenia [147]. In contrast, none of the 26 individuals, who after vaccination with an mRNA-based vaccine developed CVST, were diagnosed with thrombocytopenia. The prognosis for the ChAdOx1 nCoV-19-vaccinated individuals was inferior for CVST with 38% fatal cases, while 20% of patients who received the mRNA vaccine died [148].

### 6.2. COVID-19 Vaccine and Acute Brain Complications 

Additionally, rare cases of acute brain complications such as seizures [150], hyperacute reversible encephalopathy [151], new-onset refractory status epilepticus (NORSE) [152], delirium [153], neuroleptic malignant syndrome [154], encephalopathy [155], neurological autoimmune disease [156], and acute [157,158] and post-vaccination encephalitis have been registered after COVID-19 vaccinations [159,160,161]. In a case study, fatal acute disseminated encephalomyelitis (ADEM) was described after vaccination with the ChAdOx1 nCoV-19 vaccine based on autopsy examination and clinical medical records [162]. Another study reported the first case of encephalitis in a 48-year-old man following a booster shot with the mRNA-1273 vaccine [163]. The patient was hospitalized due to a personality change, starting with agitation and physical aggression, which evolved into mutism. According to the patient’s medical history, the transition from female to male two years earlier still required intramuscular administration of Sustanon^®^, testosterone isocaproate, every three weeks. After three days, the clinical syndromes improved, and after one week of hospitalization, the patient was released with all encephalitis symptoms gone, except for amnesia [163]. In a case series study on post-vaccinal encephalitis, the incidence of encephalitis for immunization with the ChAdOX1 nCoV-19 and the BNT162b2 vaccines was eight cases per 10 million doses (0.00008%) and two cases per 10 million doses (0.00002%), respectively [161]. This is significantly lower than the incidence of COVID-19 complications, which is 0.215% with an average mortality rate of encephalitis in COVID-19 patients of 13.4% [163].

### 6.3. COVID-19 Vaccine and Loss in the Central Nervous System 

Rare incidences of vaccine-associated myelin loss in the central nervous system (CNS) or the peripheral nervous system (PNS), including Bell’s palsy [143,164,165,166], GBS [143,155,167,168], myelitis [169,170,171,172], and multiple sclerosis have also been reported following COVID-19 vaccinations [173,174,175].

A comprehensive prospective observational analysis of 704,003 individuals receiving the first dose of the BNT162b2 mRNA vaccine in Mexico showed that among the 65.1% of the 6536 adverse events recorded of which 99.6% were non-serious, at least one had a neurologic impact [172]. Among 33 reported severe incidents, 17 (51.5%) involved the nervous system resulting in an incidence of 2.4 cases per 100,000 doses. At the time the study was published, 16 out of 17 patients had been discharged without a single fatality. Only 17 significant adverse reactions, including seven seizures, three GBS, four functional syndromes, and two transverse myelitis cases were reported [176]. 

### 6.4. COVID-19 Vaccine and Acute Transverse Myelitis

Acute Transverse Myelitis (ATM) has been presented as an unexpected common neurological adverse event in COVID-19 patients [177]. In 68% of the cases, the latency period ranged from 10 days to 6 weeks, an indication of the host neurologic complications to the virus. A short latency period of 15 h to 5 days in 32% of the cases supported the neurotropic nature of SARS-CoV-2. Three cases of ATM adverse events were reported among 11,636 individuals vaccinated with the ChAdOx1 nCoV-19 vaccine, which was exceptionally high compared to the global prevalence of 0.5 cases per million COVID-19-associated ATM cases [177]. In another approach, two cases of ATM were detected after vaccination with the BNT162b2 vaccine [178]. In one case, an 81-year-old male experienced bilateral hand weakness three days post-vaccination. In another case, tingling in the legs of a 23-year-old female was observed 21 days after vaccination. Intravenous treatment of both patients with methylprednisolone reduced their symptoms to some extent. However, the rarity of side effects of COVID-19 was disputed in a literature review, which identified 20 cases of ATM with full patient information [179]. The interval between vaccination and the onset of clinical symptoms of ATM spanned from one day to 21 days. The 20 ATM patients were treated with steroids (n = 20), plasma exchange (n = 4), or immunological adsorption (n = 1). So far, total recovery was achieved in three patients, partial recovery in sixteen patients, and an 85-year-old patient died, albeit of poor general condition 58 days post-vaccination [177]. 

### 6.5. COVID-19 Vaccine and CNS Demyelination

The association of COVID-19 vaccines with cases of CNS demyelination has been investigated in a systematic review [180]. A total of 32 cases were detected, the majority of which occurred after the administration of the first dose of an mRNA vaccine, an adenovirus-based vaccine, or an inactivated whole virus vaccine [180]. Demyelination was most frequent for mRNA-based vaccines, followed by adenovirus-based vaccines, and the least cases of demyelination were detected for inactivated vaccines. As a history of immune-mediated illnesses existed in more than half of the cases, a causal relationship between vaccination and demyelination could not be established [180].

## 7. COVID-19 Vaccines and Premature Psychiatric and Mental Disorders

### 7.1. COVID-19 Vaccines and Premature Bipolar, Depressive, and Psychotic Disorders

There is mounting evidence in the field of psycho-pathophysiology supporting the link between inflammation and its chemical mediators and the exacerbation and development of bipolar, depressive, and psychotic disorders. These findings suggest that increased inflammation (and related oxidative stress) can lead to neuropsychiatric exacerbations [18,31,44,181,182,183,184,185], and it has even been hypothesized that these three classes of neuropsychiatric diseases can be considered potential inflammatory disorders.

When it comes to vaccinations, the goal is to elicit an immune response by stimulating a pro-inflammatory reaction [186]. COVID-19 mRNA vaccines are known to initiate Th1-biased immune responses associated with increased levels of IL-2, IFN-γ, and TNF, as well as CD4^+^ and CD8^+^ T-cells [187,188,189,190]. Furthermore, the Th1 response from mRNA vaccines can activate macrophages causing further increases in IL-6 and TNF-α levels [191]. Patients with Major Depressive Disorder (MDD) are more likely to have elevated levels of TNF-, one of the three principal mediators of inflammation caused by the COVID-19 vaccinations, whereas elevated levels of IL-2 and IFN- are connected to depression [16,182]. 

The hypothesis of a potential association between COVID-19 vaccination and inflammation-triggered acute worsening of psychiatric illnesses is supported by several studies [3,192]. For example, in a case series study, a 60-year-old female patient and a 40-year-old male patient with previously well-controlled BD1 symptoms were reported to experience acute exacerbations of the disease, where the former patient developed worsened depression, mania, and psychosis within a week of receiving the second dose of the BNT162b2 COVID-19 vaccine, and the latter patient experienced depression, mania, psychosis, and suicidality after receiving the second dose of the mRNA-1273 COVID-19 vaccine, leading to changes in medication for both patients and hospitalization of the latter patient [186]. 

In another case study, a 26-year-old man with a history of Down syndrome, who had developed hypothyroidism at 10 years of age and schizophrenia at 15 years of age, was doing well and had moderately good autonomy for daily routine tasks [193]. Twenty-four hours after receiving his first dose of the BNT162b2 mRNA vaccine, he developed an acute depressive episode. The depressive symptoms resolved spontaneously after one week, but the symptoms returned and lasted for five weeks. After two days of treatment with escitalopram, the depressive symptoms diminished considerably [193]. Although rare, the psychiatric side effects of COVID-19 vaccinations need to be appropriately considered, especially in vulnerable individuals. 

### 7.2. COVID-19 Vaccines and Premature Narcolepsy 

Another neurological consequence of COVID-19 vaccinations is the development of disturbances of sleep, primarily hypersomnia, with narcolepsy being one of the primary complications of interest. Although the estimated prevalence of narcolepsy in Israel is as low as 0.14–0.21 cases per 100,000 persons [19] in comparison to 19–56 cases per 100,000 persons in the US and Europe [194], one case of a patient with a premorbid subclinical sleep disturbance that developed a clinically severe case of type 1 narcolepsy was reported after vaccination with the BNT162b2 vaccine [3]. However, as there was hardly any pre-vaccination information available regarding the health or immunological status of the patient, more information is needed before the causal relationship to COVID-19 vaccination can be confirmed [195]. 

### 7.3. COVID-19 Vaccines and Delirium

An 89-year-old patient with a history of T2DM, hypertension, stage IIIb chronic kidney disease, prostatic hyperplasia, mild hearing impairment, and depressive disorder showed signs of delirium after vaccination with the BNT162b2 vaccine, as evidenced by confusion, fluctuating attention, anxiety, and inversion of the sleep–wake cycle [21]. He used many drugs regularly, including detemir/aspart insulin, empagliflozin, bisoprolol, telmisartan, tamsulosin, and mirtazapine. COVID-19 vaccinations have been linked to sepsis-associated delirium because their detrimental effects are due to the systemic inflammatory response [194], which may induce changes in brain physiology [196]. In this patient's case, the presence of multiple delirium risk factors, such as age, polypharmacy, and sensitivity impairment, was highlighted, suggesting that the vaccine-induced immune response may have been sufficient to disrupt his weak homeostasis. 

### 7.4. COVID-19 Vaccines and Premature Neuroleptic Malignant Syndrome

Few studies have reported the development of Neuroleptic Malignant Syndrome (NMS), a potentially life-threatening neurological emergency with a mortality rate of 5.6% after COVID-19 vaccination [197]. A 74-year-old lady with dementia and bipolar illness in her medical history was brought to an intensive care unit (ICU) after developing vaccination-provoked NMS [198]. She was treated with amantadine and cyproheptadine and was discharged from the hospital after eight days. In another case study, a 61-year-old Japanese woman diagnosed with schizophrenia 35 years ago, who was recently treated with risperidone, developed NMS after receiving the BNT162b2 vaccine [154]. She was hospitalized, and the risperidone treatment was discontinued. A systemic massive infusion of fluids resulted in a decrease in creatinine levels, and she was discharged from the hospital after 10 days. In another case study, a 48-year-old male developed altered mental status, fatigue, fever, generalized weakness, and loss of appetite as a response to the initial vaccination with the BNT162b2 vaccine [17]. He was diagnosed with NMS with adrenal insufficiency, and he was hospitalized. Furthermore, he responded well to dantrolene, bromocriptine, and hydrocortisone and was discharged after 4 weeks. 

### 7.5. COVID-19 Vaccines and Acute Mania with Psychotic Features

One day after receiving the BNT162b2 mRNA vaccine, two individuals experienced bouts of intense mania with psychotic characteristics [199]. The first example is a 42-year-old man who, after receiving the first dose of vaccine, had irritation, illusions that his family was in danger, and insomnia, and was taken to the mental emergency room [199]. In the other case, a 57-year-old man with no previous history of mental illness was taken to the psychiatric emergency unit after experiencing irritability, insomnia, hearing voices, and having suicidal thoughts following his second vaccination [199]. It was postulated that the BNT162b2 mRNA vaccine might cause adverse neuropsychiatric side effects, which can be triggered either by the autoimmune mechanisms due to excessive production and release of pro-inflammatory chemokines and cytokines, particularly TNF-α, IL-1, and IL-6, or by damaging thiamine metabolism, as thiamine deficiency can trigger neuropsychiatric symptoms [199].

After receiving the mRNA-1273 vaccine, a 31-year-old man with no history of mental illness had new-onset psychosis [200]. The patient's behavior was unpredictable and odd, he had auditory hallucinations, and he had grandiose ideas that he was developing clairvoyance. The police took him to the ER, he was hospitalized, and started on antipsychotic medication. After the second dose, he experienced a dramatic worsening of his symptoms [200]. Due to the patient's already schizotypal personality traits, the vaccination may have only had a secondary impact of triggering psychosis in this individual [200]. 

### 7.6. COVID-19 Vaccines and Acute Encephalopathy and Seizures

Acute encephalopathy and autoimmune encephalitis might present neurological complications related to SARS-CoV-2 infections [201]. In the context of COVID-19 vaccines, a 32-year-old male with no significant history of psychiatric illnesses and alcohol or other substance abuse developed acute encephalopathy and seizures within a day after receiving the first dose of the mRNA-1273 COVID-19 vaccine [201]. Similar acute encephalopathy incidents with seizures were reported in individuals receiving the ChAdOX1 nCoV-19 [150,151] and mRNA-1273 COVID-19 vaccines [155]. A 68-year-old rural Indian man without comorbidities other than well-controlled arterial hypertension experienced a new onset of focal and non-motor seizures with reduced awareness and noticeable temporary behavioral changes after getting his first dose of the ChAdOx1 nCoV-19 [150]. In addition, after receiving the mRNA-1273 vaccine, severe disorientation, visual hallucinations, and a left frontal headache were detected in an 86-year-old lady who had a history of cataracts, stage III chronic renal disease, diastolic dysfunction, glaucoma, and T2DM but no history of cognitive impairment [155]. Similarly, after receiving his first dose of the mRNA-1273 vaccine, a 73-year-old man with no known neurological or psychiatric history but a history of Crohn's disease, hereditary hemochromatosis, hypertension, and hyperlipidemia was hospitalized with abrupt encephalopathy and seizures [155]. In another case report, a 77-year-old man in clinical remission with sarcoidosis and polymyalgia rheumatica developed hyperacute reversible encephalopathy after receiving the first dose of the ChAdOx1 nCoV-19 vaccine [151]. There was a significant rise in the levels of intrathecal neuroinflammatory mediators (IL-6 in both CSF and serum and IL-8 in CSF), suggesting that immunization enhanced cytokine-storm-related encephalopathy (CySE), a brain disorder associated with cytokine storm [151]. After the second dose of the BNT162b2 vaccine, a 15-year-old girl had encephalopathy, convulsions, myocarditis, and thrombocytopenia concurrently, indicating that COVID-19 vaccines may induce numerous adverse effects in some situations [202]. 

### 7.7. COVID-19 Vaccines and Premature Autoimmune Encephalitis 

A 77-year-old man with a history of coronary artery disease, hyperlipidemia, and hypothyroidism was presented to the ER with confusion, fever, and a generalized rash after receiving his first dose of the mRNA-1273 vaccine [154]. He was later diagnosed with acute aseptic meningoencephalitis and the Sweet syndrome [151]. The likelihood of a virus-mediated inflammatory response was ruled out in this case since the patient tested negative for SARS-CoV-2 infection by RT-PCR [155]. It was possible to show a temporal association between vaccination and clinical signs, but causation could not be demonstrated. In addition, the ChAdOx1 nCoV19 vaccine has been linked to a string of episodes of post-vaccine encephalitis [161]. These cases of possible autoimmune encephalitis included a 21-year-old female patient with no previous somatic or psychiatric diseases, who developed headache and progressive neurological symptoms including attention and concentration difficulties. A 63-year-old female had deep vein thrombosis in her left leg after receiving the ChAdOx1 nCoV-19 vaccine, whereas a 63-year-old male patient experienced localized aphasia and generalized twitching after receiving the same vaccine [161]. In another case series, a single-center prospective analysis of patients with neurological autoimmunity suggested a temporal association (≤6 weeks) with COVID-19 vaccinations, with a lack of other triggers. This analysis found 21 consecutive cases of neurological autoimmunity, which occurred 3–23 days after vaccinations with the BNT162b2 (n = 12), ChAdOx1 nCoV-19 (n = 8), and mRNA-1273 (n = 1) vaccines [155]. The adverse events included VITT (n = 3), CNS demyelinating diseases (n = 8), inflammatory peripheral neuropathies (n = 4), myositis (n = 3), myasthenia (n = 1), limbic encephalitis (n = 1), and giant cell arteritis (n = 1) [155]. One instance of limbic encephalitis, one case of opsoclonus myoclonus ataxia syndrome, and three cases of acute disseminated encephalomyelitis were found in a comprehensive investigation conducted in Kerala, India [203].

A 57-year-old Asian lady with hypertension was documented in a case study [203]. She had had myalgia after receiving her first dose of the ChAdOx1 nCoV-19 vaccine, and after receiving her second dose, she developed autoimmune encephalitis (AE) [204]. The low prevalence of AE (13.7 cases per 100,000 people in the general population) and the temporal relationship to the vaccination suggest that the diagnosis of vaccine-induced AE is plausible. However, definitive confirmation of causality was not possible due to the lack of suitable biomarkers and antibodies.

A 72-year-old woman with rheumatoid vasculitis was diagnosed with acute meningoencephalitis after receiving the first dose of the BNT162b mRNA vaccine [205]. Moreover, acute encephalomyelitis was diagnosed in a 55-year-old man with a history of hypertension, hyperlipidemia, and sleep apnea, after receiving the first dose of the ChAdOx1 nCoV-19 vaccine [206]. A case of autoimmune limbic encephalitis triggered by the second dose of the mRNA-1273 vaccine was detected in a previously healthy 35-year-old female [207]. A rather unique case of relapsed autoimmune encephalitis was discovered in a 72-year-old man with a history of T2DM and high blood pressure after receiving the first and the second doses of the BNT162b2 vaccine [208]. 

A 48-year-old man was diagnosed with anti-leucine-rich glioma inactivated 1 (LGI1) autoimmune encephalitis after he developed fast cognitive deterioration and hyponatremia following his second dose of the BNT162b2 mRNA vaccine [209]. In a case report, a 53-year-old male patient with first-onset generalized tonic-clonic seizures (GTCSs) and subsequent progressive neurologic symptoms including gait disturbance, dysarthria, and cognitive decline showed symptoms consistent with GABA-B receptor antibody encephalitis following a second dose of the ChAdOx1 nCoV-19 vaccine [210]. Though reports of autoimmune encephalitis following the administration of the COVID-19 vaccination are uncommon, medical professionals should be aware of the possibility and pay special attention to any potential symptoms [211].

### 7.8. COVID-19 Vaccines and Guillain–Barré Syndrome

GBS is a post-infectious autoimmune polyneuropathy that affects the peripheral nerves and nerve roots [212,213,214]. A recent cohort study with eight participating healthcare systems in the US and more than 10 million participants revealed that the incidence of GBS was elevated after receiving the Ad.26.COV2.S vaccine [215]. The unadjusted incidence rate of confirmed GBS at 1 to 21 days after receiving the Ad.26.COV2.S vaccine was 32.4 per 100,000 person-years, according to an analysis of surveillance data from the Vaccine Safety Datalink, which included 15.1 million doses of COVID-19 vaccines inoculated between 13 December 2020, and 13 November 2021 [215]. It is significantly higher than the GBS background rate of 1 to 2 per 100,000 person-years [216,217]. Furthermore, GBS appears to occur more frequently in response to immunization with the ChAdOx1 nCoV-19 and Ad26.COV2.S vaccines than with mRNA-based COVID-19 vaccines [218]. Although the number of reported actual cases of GBS after vaccination with the ChAdOx1 nCoV-19 vaccine is rather low [203,219,220,221,222,223,224], the quality of sleep might be affected in individuals with post-vaccination GBS due to persistent pain during the night [224].

### 7.9. COVID-19 Vaccines and Adult Hippocampal Neurogenesis

Although serious adverse events with COVID-19 immunizations have been a cause of concern, it has been theorized that vaccinations have protective benefits against age-related cognitive decline and mental illnesses by stimulating adult hippocampal neurogenesis (AHN) [225]. According to the grounds of this hypothesis, AHN is negatively affected by SARS-CoV-2 infection both during the acute phase of COVID-19 [226,227] and throughout the so-called protracted COVID [227]. In patients with respiratory system diseases, vaccine-induced adaptive immunity does not only block the detrimental effects of acute infection on the AHN but may also promote enhanced AHN [227]. This is further supported by observations of the potential neurotrophic effects of vaccines against respiratory tract infections, such as influenza and tuberculosis [228,229,230,231]. Curiously, these beneficial effects are linked to the vaccination-induced elevated levels of anti-inflammatory cytokines, such as IFN-γ, IL-4, and TGF-β (i.e., molecules potentially linked to acute exacerbations of neuropsychiatric diseases; see above), which can promote proliferation and differentiation of neural stem cells (NSCs) [4,226,228,229].

## 8. COVID-19 Vaccines and Premature Autoimmune Spectrum Disorders

Vaccinations against COVID-19 have been associated with a small number of rare side effects, also reported for autoimmune disorders [232]. The specific mechanisms through which COVID-19 vaccines may trigger autoimmune reactions are unknown. The COVID-19 vaccine has been related to an increase in the occurrence of blistering illnesses such as bullous pemphigoid [22]. After COVID-19 immunization, there was a significant clonality of T cells reacting to SARS-CoV-2-associated epitopes in lesions from patients with new-onset bullous pemphigoid, which was not the case before COVID-19 mass immunizations. This implies that the immunization induced a long-lasting adaptive immune response, which fueled lesion development. As a result, COVID-19 vaccines may induce unanticipated T-cell responses, leading to the establishment of new autoimmune diseases or triggering flares in those who already suffer from autoimmune diseases [23].

Molecular mimicry has been proposed as the fundamental concept to explain the emergence of occasional adverse events in vaccinated individuals. This theory postulates that adjuvants (such as virosomes, aluminum salts, immune modulatory complexes, oil-in-water emulsions, montanide, squalene, lipovant, and xenobiotic adjuvants) accompanying vaccines share similar features with self-antigens. One possibility is that "innocent bystanders” are activated, which results in autoreactive T cells, polyclonal activation, and epitope dissemination [233]. 

Anti-SSA/Ro antibody- and hypocomplementemia-associated severe autoimmune thrombocytopenia is one of the several reported autoimmune diseases [234]. A comprehensive literature assessment on autoimmune disorders emerging after COVID-19 vaccinations revealed a total of 276 reports [235]. GBS and VITT patients were the most common cases, leading to hospitalization. There have also been some reports of autoimmune liver disease (n = 8), immune thrombocytopenic purpura (n = 7), IgA nephropathy (n = 5), autoimmune polyarthritis (n = 2), rheumatoid arthritis (nm = 2), Graves' disease (n = 4), and systemic lupus erythematosus (n = 3). Despite the appearance of autoimmune disorders in individuals receiving one or two doses of COVID-19 vaccines, the cases are rare, and the undeniable benefits of vaccination remain strong [235]. Patients in China with autoimmune rheumatic disorders (ARDs) developed an increase in their condition after receiving an inactivated COVID-19 vaccine [236]. There were a total of 1507 patients with ARDs who participated in this study: 614 had systemic lupus erythematosus (SLE), 434 had rheumatoid arthritis (RA), 122 had Behçet’s disease, 76 had psoriatic arthritis/psoriasis, and 74 had primary Sjögren syndrome. The remarkable tolerance of administration of the inactivated COVID-19 vaccine in the cohort with autoimmune rheumatic conditions demonstrated that disease was induced in only 158 individuals (10.5%), and none of the cases were fatal [236]. The COVID-19 database study was funded by the European League Against Rheumatism (83% of mRNA vaccinations). 37% of the 5121 study participants reported vaccine-related side effects, and 4.4% developed rheumatic illnesses. Inflammatory manifestations of the musculoskeletal system have been documented. These include enthesis, inflammatory spinal pain, synovitis, tenosynovitis, and discomfort/stiffness with serological evidence of inflammation four weeks after receiving one of the COVID-19 vaccines (BNT162b2, mRNA-1273, ChAdOx1 nCoV-19, or Ad26.COV2.S). Sixty-six patients took part in the trial. There were polymyalgia-rheumatica-like symptoms exhibited in 41% of patients, oligoarthritic in 32%, and polyarthritis in 27% [237]. After receiving his third dose of the inactivated COVID-19 vaccine, a 77-year-old male patient developed an eruptive skin disease [238]. Seven days after receiving her first dose of the ChAdOx1 nCoV-19 vaccine, a 62-year-old Asian lady developed a non-blanching, petechial rash on both of her lower limbs [238,239]. In addition, to her headaches, she also experienced chronic muscular and joint discomfort. These two instances, as well as previous reports of vasculitis and autoimmune syndromes after receiving COVID-19 injections, suggest a possible connection between an “adjuvant” included in the vaccine and the development of such disorders in genetically and immunologically vulnerable individuals [240,241]. This might explain why some persons have flares or new onsets of autoimmune illnesses after receiving a COVID-19 vaccine, as it has been hypothesized that age-associated B cells contribute to the immunological response elicited by these vaccinations. Although reports of systemic autoimmune reactions following administration of COVID-19 vaccinations have been made, a causal relationship between the two cannot be established at this time.

## 9. COVID-19 Vaccines and Other Premature NCDs 

Although systemic transient adverse events such as fever, headache, widespread fatigue, muscular pain, and nausea are common after COVID-19 vaccinations [21], their description is not included in this review. However, SARS-CoV-2 infections have been linked to several eye conditions, including conjunctivitis, scleritis, orbital inflammatory disease, phlyctenular keratoconjunctivitis, and retinal involvement [239]. Related to mass vaccinations against COVID-19, potentially harmful adverse events have been reported [239,240,241,242,243]. For example, unilateral edema most noticeable on the upper eyelid was detected in three women receiving the BNT162b2 vaccine [243]. The three patients, subjected to treatment with antihistamines, oral steroids, or only kept for observation, respectively, all showed complete improvement in their orbital swelling within 48 h [243]. Furthermore, three other individuals who received the BNT162b2 vaccine experienced ecchymotic or purpuric sores on their upper eyelids 1–3 weeks after immunization [244]. Moreover, two individuals showed symptoms of clots in their superior ophthalmic vein. A 55-year-old female receiving the ChAdOx1 nCoV-19 vaccine exhibited conjunctival infection, retro-orbital discomfort, and diplopia seven days post-vaccination [245]. A 45-year-old male experienced left eye discomfort, increased ptosis, reduced eyesight, and binocular diplopia 7 days after receiving the mRNA-1273 COVID-19 vaccine [246]. Complete ophthalmoplegia and an afferent pupillary deficit were observed in the patient.

In the context of uveitis, based on a one-year retrospective systematic review, it took an average of 8 days (standard deviation: 8.6) for ocular symptoms to appear in 34 individuals following the administration of COVID-19 vaccines [247]. A total of 10 individuals (29.4 %) showed symptoms in both eyes. A mean visual acuity of logMAR 0.421 0.455 (20/52 in Snellen notation) was found in 40 eyes [247]. In another study, a 34-year-old man showed ocular discomfort (OD) and nasal redness, as well as a floater in the right eye, followed by a rapid decrease in his vision over the course of four days after administration of the ChAdOx1 nCoV-19 vaccine [248]. Similarly, a 50-year-old Chinese woman experienced bilateral impaired vision 5 days after receiving an inactivated COVID-19 vaccine [249]. Her foveal reflex was nonexistent, her optic disc was pallid, and she had macular edema. Bilateral choroiditis was supported by fluorescein angiography findings [249]. In another case study, a 43-year-old woman with a 6-year history of Vogt–Koyanagi–Harada (VKH) disease showed additional intermittent infliximab infusions [250]. The patient had a significant resurgence of illness 6 weeks after the second BNT162b2 vaccine dose. Moreover, a 71-year-old male immunized with the ChAdOx1 nCoV-19 vaccine showed reactivation of herpes zoster [251]. The symptoms of the patient comprised panuveitis, circumcorneal congestion, multiple fine keratic precipitates (KPs), virtutis, and extensive acute retinal necrosis. 

In another case study, a 48-year-old male experienced progressive bilateral corneal melting 5 weeks after receiving his first dose of the ChAdOx1 nCoV-19 vaccine [252]. Ultrasonography revealed bilateral extensive choroidal separation, uveal tissue prolapses, and widespread conjunctival and ciliary congestion [252]. Following immunization, there is a risk of compromised corneal graft due to several different processes [253]. For example, the native-state immunologic privilege of the cornea may be jeopardized by increased vascular permeability induced by vaccination. Graft edema, as seen in this study, lends credence to this hypothesis. Since organ transplants often lead to increased expression of MHC antigens following rejection, it was postulated that vaccination could trigger the expression of corneal MHC [253]. Donor cells are a potential sitting duck for the host immune system because they lack MHC expression. In the context of vaccination against COVID-19, it has been hypothesized that SARS-CoV-2 RNA is present in the aqueous humor of individuals with asymptomatic infections, hence providing an additional pathway for corneal transplant rejection [254]. For example, corneal tissue from COVID-19 asymptomatic donors showed the presence of SARS-CoV-2 RNA in four out of 101 samples, suggesting rather prolonged detection of RNA as no active replicative virus was found [254,255]. As a result, immunization against COVID-19 during a current or recently asymptomatic infection might result in huge amounts of antibody–antigen complexes, which could cause severe inflammation and jeopardize corneal transplant success.

## 10. Effects of COVID-19 Vaccines on Reproductive Health and Lactation

The effects of COVID-19 vaccinations on reproduction-related functions such as menstruation and fertility have been of concern. Several surveys including a collection of data from the VAERS have indicated potential safety concerns related to menstrual disorders in COVID-19-vaccinated young adult females [25]. Furthermore, in a retrospective study including 408 women, SARS-CoV-2 infections and COVID-19 vaccinations were indicated to influence the menstruation cycle and cause alterations to it [256]. In a large study of 14,153 women, 78% reported changes in their menstrual cycle after COVID-19 vaccination, although the changes were generally premenstrual and mild [257]. In another study of 219 women vaccinated with the BNT162b2 mRNA vaccine, relatively high rates of irregular bleeding and menstrual changes were reported [258]. However, it needs to be pointed out that menstrual changes have been reported for both adenovirus- and mRNA-based COVID-19 vaccines [259] indicating that the effects are likely the result of immune responses after vaccination. This has also been confirmed for vaccination against human papilloma virus (HPV) [260]. Moreover, it has been documented that SARS-CoV-2 infections, although not generating changes in sex hormone concentrations, caused menstrual volume decrease and cycle prolongation [261]. Additional recent studies in the US [262] and Norway [263] have been conducted. In the former study, six cycles were monitored in 3959 American women showing no effect on the timing of the subsequent period after the first vaccine dose, while a delay of 0.45 days was registered after the second dose [262]. The study of 5688 Norwegian women demonstrated that 37.8% of participants had at least one specific menstrual change from normal although similar findings were reported in pre-vaccination cycles [263]. In summary, vaccine-induced changes to the menstrual cycle seem to be short-lived [259] and they are generally small compared to natural variation. They are also quickly reversible [259].

### 10.1. COVID-19 Vaccines and Female Infertility

In the context of fertility, there has been public concern about COVID-19 vaccines causing female infertility [26]. This outcry is based on misinformation and erroneous interpretation of data from clinical trials, where pregnancy rates were extremely low due to participants using contraception [264] or the services of fertility clinics [265]. For example, it has been confirmed that claims that the similarity between syncytin-1 and the SARS-CoV-2 spike protein present in many of the COVID-19 vaccines would cause female sterility by induction of immune cross-reactivity are unfounded [266]. Using frozen embryos as a model for the comparison of implantation rates in COVID-19-vaccinated, COVID-19-positive, and COVID-19-negative women, no differences were observed [266]. In a review on male and female fertility, it was indicated that in both men and women, no fertility problems existed and no increase in adverse pregnancy occurred after SARS-CoV-2 infection or COVID-19 vaccination [267]. Furthermore, the transfer of maternal antibodies through the placenta vaccination can provide clear benefits. In a reproductive toxicity study, rats were intramuscularly immunized with 30 μg/mL BNT162b2 mRNA vaccine, equivalent to a dose 300 times higher than used in human mass vaccinations [268]. Robust neutralizing antibody responses were detected in animals before mating, at the end of gestation and lactation, and also in the offspring. Female mating, ovarian or uterine parameters, embryo–fetal postnatal survival, growth, physical development, or neurofunctional offspring development were shown to be unaffected by COVID-19 vaccinations [268]. Despite multiple comments on social media platforms that COVID-19 vaccines pose negative effects on fertility, there is no scientific evidence of such cases [269]. There is today no link or any theoretical reason why any of the available COVID-19 vaccines might affect fertility. For this reason and, additionally, knowing that pregnant women are at a higher risk of hospitalization or death after contracting COVID-19, it is highly advisable for individuals planning pregnancy to be fully vaccinated [269].

### 10.2. COVID-19 Vaccines and Male Infertility

In the context of male fertility, the risk of testicular damage by COVID-19 has been investigated [270]. Moreover, it has been hypothesized that secondary immunological and inflammatory levels elevated during severe viral infection in the testicles, could potentially damage testicular cells and could even cause infertility [269]. Furthermore, several studies have indicated that the COVID-19 pandemic has affected sexual function and sexual activity [271]. In the context of COVID-19 vaccines, 45 volunteers aged 18–50 years were evaluated for sperm parameters before and after vaccination with BNT162b2 or mRNA-1273 vaccines [272]. After two mRNA vaccine doses, no significant decrease was observed in any of the sperm parameters. Similarly, the vaccination of 72 patients with the BNT162b2 vaccine showed no change in sperm parameters in men with normal or abnormal semen production, which is reassuring due to the on-going global mass vaccination [273]. 

### 10.3. COVID-19 Vaccines and Lactation

One area of interest and concern comprises the effect of vaccination on lactation and the potential presence of traces of vaccine-related SARS-CoV-2-specific antibodies and/or mRNA in breast milk [27]. In this context, no SARS-CoV-2 RNA was detected in 37 milk samples from 18 breastfeeding COVID-19 patients measured by RT-qPCR [149]. In contrast, 62% of COVID-19-positive breast milk samples had IgA and IgG antibodies specific to SARS-CoV-2 and were able to neutralize SARS-CoV-2 infectivity in vitro. This indicated that breast milk does not facilitate mother-to-infant SARS-CoV-2 transmission but is a good source of neutralizing activity against SARS-CoV-2 in the form of IgA and IgG. In another investigation, plasma and milk samples were collected from 22 nursing staff members before, during, and after immunization with mRNA-based vaccines (BNT162b2, mRNA-1273) [274]. Breast milk and plasma were shown to have high concentrations of IgA and IgG against SARS-CoV-2. In a comparable investigation, 50 nursing mothers were tested before and after vaccinations with the BNT162b2 and mRNA-1273 vaccines (before the second dose and 4–10 weeks after the second dose) [275]. Two women and two newborns tested positive for COVID-19. However, no serious adverse effects were seen in either the mothers or the infants. Maternal plasma showed a rise in anti-SARS-CoV-2 IgG and IgM antibodies, while milk samples showed an increase in anti-SARS-CoV-2 anti-RBD IgA and IgG antibodies [275]. In addition, 10 nursing mothers had samples of their milk and serum taken 20 days after receiving their first dose of the BNT162b2 vaccine and 7 days after receiving their second dose [276]. All serum samples tested positive for anti-SARS-CoV-2 S antibodies after the initial dose, but only two milk samples did. All blood and milk samples collected after the second dose were positive for anti-SARS-CoV-2 S antibodies [276]. These data suggested that immunization of nursing mothers might protect newborns from SARS-CoV-2 infections.

In another study involving seven breastfeeding mothers vaccinated with BNT162b2 or mRNA-1273 vaccines, milk samples were collected 4 to 48 h post-vaccination and total RNA was isolated and RT-qPCR was performed for the evaluation of the presence of SARS-CoV-2 RNA in milk [277]. In the 13 samples analyzed, no SARS-CoV-2 RNA was detected. However, in a response letter to the study, it was pointed out that these results should not be generalized and additional studies are required to confirm the findings [278]. Recently, in a cohort study of 11 breastfeeding women receiving either the BNT162b2 or the mRNA-1273 vaccines, milk samples were collected before the vaccination and up to 5 days post-immunization [279]. Trace amounts of BNT162b2 and mRNA-1273 RNA were detected in seven samples from five different individuals at various time points up to 45 h (2 days) after vaccination. However, no vaccine-related RNA was detected pre-vaccination or beyond 48 h post-vaccination [279]. The small sample size and the lack of functional studies to determine whether the detected vaccine-related mRNA is translationally active need to be addressed in larger studies. In any case, the authors stated that breastfeeding is safe after maternal COVID-19 vaccination although additional safety studies are requested concerning breastfeeding during the first 48 h after vaccination [279].

## 11. COVID-19 Vaccines and Hesitancy 

Since the advent of vaccine development and vaccinations, skepticism related not only to efficacy but also to safety has been a commonly visited topic. Not surprisingly, the COVID-19 pandemic with the unprecedented rapid vaccine development and the application of novel strategies including mRNA-based vaccines has been no different, and even worse. As described above, adverse events have been registered for various indications, which is not that surprising due to the huge number of vaccinations carried out globally with 62.8% of the world population receiving at least one COVID-19 vaccine dose and a total of 12.8 billion doses administered (Coronavirus (COVID-19) Vaccinations—Our World in Data, https://ourworldindata.org/covid-vaccinations, accessed on 13 October 2022). 

Overall, the adverse events and death rates in individuals immunized with COVID-19 vaccines are significantly lower than in unvaccinated persons demonstrating that the benefits are outweighing the risks by a large margin. When 12.8 billion doses have been administered, it is no surprise that adverse events occur, especially taking into account that many individuals carry pre-existing conditions and comorbidities, which can have a negative impact on the outcome of vaccination safety.

Several surveys and systematic reviews on COVID-19 vaccine hesitance have been conducted. For example, a PubMed database search conducted on publications until 17 July 2021, showed a broad variation of vaccine acceptance from 12 to 91.4% [280]. A correlation between unwillingness toward COVID-19 vaccination and Black/African American origin was established. Moreover, sex, age, race, education level, and income status showed a strong influence on low or high approval of COVID-19 vaccinations. In the US population, the highest vaccine hesitancy was reported in Black/African Americans and in pregnant or breastfeeding women, while low vaccine hesitancy was seen in males [280]. In a national cross-sectional on-line survey conducted in August 2021 in China covering a total of 29,925 residents, the overall prevalence of COVID-19 vaccines was only 8.40% for the first-dose vaccination and 8.39% for the booster vaccination [281]. Moreover, the willingness to be vaccinated was higher for women, persons with a higher educational level, married people, people in good health, non-smokers, and persons responsive to precautious hygiene (washing hands, wearing masks, social distancing, etc.). Individuals with a lower rate of belief in conspiracy theories and a higher level of trust in medical doctors favored vaccinations. Among youth aged 12–15 years, 42% were not hesitant at all regarding COVID-19 vaccines, 22% were “a little hesitant”, 21% were “somewhat hesitant, and 15% were “very hesitant” according to a study from December 2021 [282]. No statistically significant differences were discovered related to age, gender, race, and parental education. However, a statistically significant correlation between vaccine hesitancy and TV watching was reported. In another comprehensive global assessment of 76,471 healthcare workers, a broad spectrum of vaccine hesitancy from 4.3% to 72% was recorded [283]. The meta-analysis of 35 studies showed remarkable differences in vaccine hesitancy in different countries. According to the publication, the lowest hesitancy of 4.3% was reported in China. In the US, hesitancy varied between 8% and 18%. Across Europe, the range was from 7% and 32.5% with the highest hesitancy seen in Greece and France. Not surprisingly, vaccine hesitancy was very high in Africa with the highest number of 72% registered in Congo. Globally, the general concerns were related to vaccine safety, efficacy, and potential side effects. The studies also revealed that male healthcare workers, persons of older age, and doctoral degree holders were more favorable toward COVID-19 vaccinations. Moreover, the acknowledgment of getting infected by SARS-CoV-2, direct care for patients, and experience with influenza vaccination increased the acceptance of COVID-19 vaccines. In another study of 4571 Norwegian adults, several subgroups hesitant toward COVID-19 vaccination were identified [284]. These included males, residents in rural areas, and parents with children younger than 18 years. Moreover, individuals preferring information from peers, social media, on-line forums, and blogs were more prone to hesitancy. The study also called for additional strategies to reduce vaccination fears and campaigns targeting the spread of false information [284]. In an editorial, Storey stressed the importance of access to accurate information related to COVID-19 vaccines and vaccinations and the necessity of counteraction against misinformation [284]. In another study based on an on-line survey with 467 participants, a positive correlation between conspiracy theories and vaccine hesitancy was detected [285]. Moreover, individuals with a low fear of COVID-19, including SARS-CoV-2/COVID-19 denial, showed vaccine hesitancy. It was concluded that vaccine hesitancy puts public health at risk, and it is important to understand the psychological factors behind it to be able to address and correct the situation through the spread of accurate information. Finally, in a US national survey, it was demonstrated that individuals who considered the COVID-19 vaccines unsafe knew less about SARS-CoV-2/COVID-19 and were more likely to believe in COVID-19 vaccine myths and conspiracy theories [286]. They were also less educated, had a lower income, and lived in rural areas. Overall, the common message is that it is of the utmost importance to openly communicate with the public and to provide genuine accurate information on COVID-19 vaccines and their clear and undisputed benefits compared to the risks of vaccination and contracting COVID-19 with its complications.

## 12. Conclusions

This article provides an overview of the probable association between NCDs and COVID-19 vaccines (Table 1). 

As a result of the global administration of more than 12 billion COVID-19 vaccine doses (Coronavirus (COVID-19) Vaccinations—Our World in Data, retrieved on 13 October 2022), adverse effects are expected. However, despite the likelihood that severe hyperglycemia is exceedingly uncommon in COVID-19 vaccine recipients, it is essential for clinicians to be aware of these side effects and anticipate severe hyperglycemia in individuals exhibiting post-vaccination symptoms such as excessive urination, excessive thirst, vision problems, and fatigue [41]. It is therefore appropriate that patients encountering hyperglycemic symptoms after COVID-19 vaccinations undergo early examination and continuous monitoring [113]. Although the association between COVID-19 vaccines and newly diagnosed diabetes has been noted, it is not yet known if this is a causative relationship or perhaps a coincidence. As successful immunization efforts have decreased hospitalizations and deaths caused by COVID-19 [287,288], there is no question that the advantages of vaccination outweigh its risks. 

In the context of CVDs, since a causal relationship has not been demonstrated between COVID-19 vaccination and the onset of hypertension, and patients with hypertensive crisis rarely need hospitalization, COVID-19 vaccinations show substantial benefits compared to its risks. Both adenovirus- [8,77] and mRNA-based [85] COVID-19 vaccines may be associated with rare cases of VITT. Regarding arrhythmia, although a case of paroxysmal ventricular arrhythmia was reported for the BNT162b2 vaccine, no causal association was found. Moreover, exposure to COVID-19 presents a significantly higher risk of developing arrhythmia [93]. The association between the BNT162b2, ChAdOx1 nCoV-19, or inactivated COVID-19 vaccines and myocardial infarction has also been suggested. The trend of COVID-19-vaccine-related myocarditis, on the other hand, is comparable to that of other viral infections, with a greater prevalence among teenagers and young adult males [103,107]. Most notably, the risk of myocarditis and hospitalization in this group of vaccinated individuals is lower than that in unvaccinated COVID-19 patients [109]. 

In terms of kidney disorders, AKI, AKD, proteinuria, edema, gross hematuria, and other renal adverse effects, a few case reports of hospitalization have been reported after COVID-19 vaccinations [109]. 

Regarding neurological disorders, mass vaccinations have confirmed that the risks of acquiring severe neurological complications are by far much lower for individuals vaccinated with COVID-19 vaccines than individuals who tested positive for COVID-19. Among all vaccine-associated neurological adverse events, only ATM was not classified as rare, as three ATM adverse events were discovered among 11,636 individuals vaccinated with the ChAdOx1 nCoV-19 vaccine [177]. 

An association between COVID-19 vaccinations and inflammation-triggered acute aggravation of psychiatric diseases in terms of psychiatric conditions and mental disorders [186,193] have been reported. Therefore, monitoring fragile individuals is important. Similarly, the medical community should pay attention to the rare cases of adverse events occurring for autoimmune encephalitis after COVID-19 vaccination.

The effect of COVID-19 vaccination on reproductive health and lactation has received plenty of attention [25,26,27]. No permanent impact of COVID-19 vaccines on reproduction-related processes such as menstruation, and female and male fertility, except for temporary disturbances in the menstruation cycle and minor drops in sperm counts immediately after vaccinations, have been found in the literature. In the context of breastfeeding, vaccination of lactating women has been considered safe.

Since the introduction of vaccines and immunizations, doubt over both safety and efficacy has been frequently discussed. The COVID-19 pandemic, with its unprecedentedly quick vaccine development and use of cutting-edge technologies, and the worldwide mass vaccination process, has elevated vaccine skepticism and hesitancy to unseen levels. Given the massive number of immunizations performed globally—62.8% of the world population has received at least one COVID-19 vaccine dose, and a total of 12.8 billion doses have been administered—it is not surprising that adverse events occur for NCDs. Moreover, although vaccinated people have tested positive for SARS-CoV-2, vaccinations significantly reduced the severity of COVID-19, and decreased hospitalization and death rates have been recorded compared to unvaccinated individuals. Overall, based on the literature, adverse events and fatality rates in people inoculated with COVID-19 vaccines are rare and much lower than in unvaccinated people; confirming that the benefits far exceed the dangers of vaccination. However, communication of accurate information in terms of scientific discoveries must be shared with the general public to underline the benefits of vaccinations, and also to honestly discuss any adverse events related to NCDs. It is also necessary to continue investigations on the potential negative consequences of COVID-19 vaccinations, determine whether they are incidental or causal, and take the necessary actions to optimize vaccine safety.

## Figures and Tables

**Table 1:** Summary of findings and incidences related to various COVID-19 vaccines.

Vaccine	Findings, Comments	Incidence	Ref.
**Diabetes**			
ChAdOx1 nCoV-19	1 case of DKA	Unclear temporal or causal	[10]
Covaxin	1 case of DKA	Unclear temporal or causal	[10]
mRNA-1273	1 case of DKA, unclear temporal or causal	Disease related (genetics)	[11]
ChAdOx1 nCoV-19	3 cases of hyperglycemia	COVID-19 related	[37]
BNT162b2	1 case of HHS	Disease related (genetics)	[44]
mRNA-1273	Phase III: 1 SAE in 487 T1DM patients	Not related to vaccine	[49]
**Cardiovascular diseases**			
mRNA-1273	23 cases of myocarditis, although rare as >2 million vaccinations	Vaccine related	[99]
mRNA-1273, BNT162b2	VigiBase: 8664 AEs, 1251 potentially associated with mRNA vaccines	Potentially associated with vaccines	[100]
BNT162b2	2.5 million persons at least one dose	COVID-19 related	[101]
**Acute kidney disease**			
mRNA-1273	ANCA after second dose	Vaccine related	[16,141]
BNT162b2	ANCA after second dose	Vaccine related	[16,141]
**Neurological disorders**			
ChAdOx1 nCoV-19	8 cases of encephalitis per 10 million	COVID-19 and vaccine related	[177]
BNT162b2	2 cases of encephalitis per 10 million	COVID-19 and vaccine related	[177]
**Psychiatric and Mental Disorders**			
Ad26.COV2.S	Enhanced incidence of GBS	COVID-19 and vaccine related	[241]
**Autoimmune Disorders**			
ChAdOx1 nCoV-19	1 case of non-blanching, petechial rash	Vaccine related	[238,239]
**Other premature NCDs**			
BNT162b2			
	3 cases of unilateral edema, healed after 48 h	Vaccine related	[243]
	3 cases of purpuric sores	Vaccine related	[244]
	2 cases of clots in superior ophthalmic vein	Vaccine related	[244]
	1 case of conjuctival infection	Vaccine related	[245]
ChAdOx1 nCoV-19	Reactivation of herpes zoster	Vaccine related	[251]
mRNA-1273	1 case of ptosis and binocular diplopia	Vaccine related	[246]

## Data Availability

Not applicable.
